# Acoustoelectric current in graphene nanoribbon due to Landau damping

**DOI:** 10.1038/s41598-021-95896-6

**Published:** 2021-09-09

**Authors:** K. A. Dompreh, K. W. Adu, D. Sakyi-Arthur, N. G. Mensah, S. Y. Mensah, A. Twum, M. Amekpewu

**Affiliations:** 1 Department of Physics, College of Agriculture and Natural Sciences, University of Cape Coast, Cape Coast, Ghana; 2 Department of Physics, Pennsylvania State University-Altoona College, Altoona, PA 16601 USA; 3 Material Research Institute, Pennsylvania State University, University Park, PA 16802 USA; 4 Department of Mathematics, College of Agriculture and Natural Sciences, University of Cape Coast, Cape Coast, Ghana; 5 Department of Applied Physics, C. K. Tedam University of Technology and Applied Sciences, Navrongo, Ghana

**Keywords:** Two-dimensional materials, Nanoscale materials, Electronic properties and devices

## Abstract

We perform self-consistent analysis of the Boltzmann transport equation for momentum and energy in the hypersound regime i.e., $ql \gg 1$ ($q$ is the acoustic wavenumber and *l* is the mean free path). We investigate the Landau damping of acoustic phonons ($LDOAP$) in graphene nanoribbons, which leads to acoustoelectric current generation. Under a non-quantized field with drift velocity, we observed an acoustic phonon energy quantization that depends on the energy gap, the width, and the sub-index of the material. An effect similar to Cerenkov emission was observed, where the electron absorbed the confined acoustic phonon energy, causing the generation of acoustoelectric current in the graphene nanoribbon. A qualitative analysis of the dependence of the absorption coefficient and the acoustoelectric current on the phonon frequency is in agreement with experimental reports. We observed a shift in the peaks when the energy gap and the drift velocity were varied. Most importantly, a transparency window appears when the absorption coefficient is zero, making graphene nanoribbons a potential candidate for use as an acoustic wave filter with applications in tunable gate-controlled quantum information devices and phonon spectrometers.

## Introduction

Landau damping of plasma waves (*LDOPW*) is the loss of energy from the collective motion of plasma waves to individual particles. This causes plasmons to decay by exciting an electron below the Fermi level^1,2^. The mechanism of *LDOPW* has been observed in various systems, such as plasma oscillations (Langmuir waves) and accelerators^3^. In semiconductors, Landau damping of acoustic phonons (*LDOAP*) occurs in the hypersound regime during electron–phonon interactions^4, 5^. This has been studied using Raman spectroscopy^6^, and high-resolution electron-energy-loss spectroscopy (*HREELS*)^7^.

The interaction of acoustic phonons with charge carriers in bulk semiconductor materials causes an amplification of acoustic waves and was predicted by Tolpygo and Uritskii in 1956^8^. This phenomenon leads to an absorption or amplification of acoustic phonons^9^, as observed in the acoustoelectric effect (*AE*)^10^, acoustomagnetoelectric effect (*AME*)^11–13^, acoustothermal effect (*ATE*)^14^ and acoustomagnetothermal effect (*ATME*)^15, 16^. The mathematical relation between absorption coefficient (${\Gamma }_{{\varvec{q}}}$) and the acoustoelectric current ($J_{ac}$) was presented by Weinreich^17^ as a ratio of $J_{ac} /{\Gamma }_{{\varvec{q}}}$ and confirmed experimentally in n-type germanium by Pomerantz^18^. These effects have been studied theoretically in semiconductor superlattices (*SSLs*)^10, 19, 20^ and confirmed experimentally in GaAs/AlGaAs^21, 22^ and GaAs/LiNbO_3_^23^ SSLs. Azizyan^25^ calculated the absorption coefficient in a quantized electric field, while Shmelev and Zung^24^ calculated the absorption coefficient and renormalization of the short-wave sound velocity. Cerenkov emission (CE) is one of the most commonly used methods for investigating acoustic effects^28, 29^. When a Non-quantized electric field (*E*_*D*_) with a drift velocity ($v_{D}$) is applied to a material and the drift velocity exceeds the velocity of sound ($v_{D} > v_{s}$), amplification of acoustic phonons occurs, whereas absorption of the acoustic phonons occurs when $v_{D} < v_{s}$. Vyazovsky et al.^26^ and Bau et al.^27^ studied the intraband absorption of electromagnetic wave in *SSLs*. Mensah et al.^30^ theoretically proposed the amplification of acoustic phonons via *CE* in *SSLs*, which was confirmed experimentally by Shinokita et al., where they achieved a 200% increase in the amplification of acoustic phonons. This phenomenon has been demonstrated to lead to sound amplification by stimulated emission of radiation (SASER)^28^.

In low-dimensional structures, the motion of surface acoustic wave (*SAW*) in the hypersound region is described as quantized lattice vibrations or surface phonons, which typically extend to 10^13^ Hz. *SAW* is generated by the deformation of the material caused by the intraband transitions of electrons under an applied field^31^. Theoretical and experimental studies of acoustic wave effects in graphene^32–35^, quantum wells (*QWs*)^36^, carbon nanotubes^37–41^, and rectangular quantum wires^42^ have been investigated in the megahertz (*MHz*), the gigahertz (*GHz*) and the terahertz (*THz*) regions. Thalmeier et al.^43^ observed Landau oscillations as a function of gate voltage in graphene. Zhang et al.^44^ obtained a strong absorption when the carrier density and the field were increased as a result of electrons colliding with the acoustic phonons under a drift electric field. Such interaction can also generate sound waves. Considering acoustic phonons as quantized sound waves of frequency ($\omega_{q}$), the conducting electrons can absorb the sound energy. This leads to damping of the acoustic phonons and, subsequently, the production of acoustoelectric current. This form of damping is referred to as Landau damping of acoustic phonon due to Cerenkov emission (*LDOCE*). *LDOCE* occurs when the drift velocity is less than the speed of sound in a material. The absorption of the phonon energy is determined by the energy balance of the system. As the frequency of the acoustic phonon increases, the absorption also increases, until there is a resonance beyond which the absorption decreases. This phenomenon has been observed in several graphene-based *AE* experiments and has been used in the fabrication of sensing devices such as humidity sensors^46^, photodetectors^47^, and gas sensors^48^.

The quantum Hall effect is observed when sound waves in a material are subjected to magnetic fields^45, 56^. For low fields, a large attenuation occurs when the frequency of the sound is an integral multiple of the cyclotron frequency. At high fields, oscillatory attenuation resulting from geometric resonance occurs when the wavelength of sound is an integral or half-integral multiple. According to Zhang et al.^44^, the absorption in graphene depends strongly on temperature and can be adjusted by changing the carrier density, suggesting the influence of doping on the absorption of acoustic phonons. That is, doping or patterning single-layer graphene (*SLG*) into *GNRs* creates a tunable multiband absorption effects, which opens an energy gap ($\Delta$) that varies with the width (*N*), the quantized wave vector ***B*** and the electron momentum. ***B*** varies with the width of the *GNR*, the lattice constant ($a_{c - c}$) and the sub-band index (*Pi*). To the best of our knowledge, there has been no theoretical investigation of *LDOCE* in *GNR* even though some experimental evidence of the phenomenon exists^35^. Poole et al.^35^ reported a nonlinear *AE* in *GNR* of width $N < 500 \;{\text{nm}}$ when stimulated with a *DC* current. They observed a resonance between the measured current maxima, after which the current decreased. Liang et al.^48^, Zheng et al.^49^ and Okuda et al.^50^ reported similar behavior, where acoustic charge transportation was induced by *SAW* propagation in the graphene. Morgado et al.^51^ also reported negative Landau damping in bilayer graphene, where they measured a DC electric current induced by a static voltage across the graphene sheet. In this work, we study the nonlinear *AE* in *SLG* and *GNR-500* with degenerate energy dispersion in the hypersound regime. We adopted the Boltzmann kinetic equation for the electron system interaction with the sound waves and calculate the absorption coefficient ($\Gamma_{q}$) for the acoustic phonon population $(N_{q} (t))$. The phonon dynamics are assumed to distort the electron distribution function (*f*_*k*_), thus affecting those electrons whose velocities in the direction of sound propagation are close to the sound velocity. Herein, we theoretically examine the effect of *DC* fields on *LDOCE* in an *SLG*/*GNR-500.*

## Methods, results, and discussion

To gain insight into *LDOAP*, we analyzed the effect of electron–phonon interactions in a gate-controlled single *SLG* and *GNR-500*. An in-plane current *I* was applied along the x-direction of the graphene sheet, which was biased by the presence of the source-to-drain voltage $v_{sd}$. The electronic transition rate induced by electron interaction with acoustic phonons is given by the kinetic equation for the acoustic phonon population $N_{q} \left( t \right)$ ^10, 19, 29, 52^, expressed as:

1 $$\begin{gathered} \frac{{\partial N_{q} }}{\partial t} = \frac{2\pi }{\hbar }g_{s} g_{v} \mathop \sum \limits_{{k,k^{\prime}}} \left| {C_{q} } \right|^{2} \delta_{{k,k^{\prime}}} \left\{ {\left| {N_{q} \left( t \right) + 1} \right|f_{k} \left( {1 - f_{{k^{\prime}}} } \right)\delta \left( {\varepsilon_{{k^{\prime}}} - \varepsilon_{k} + \hbar \omega_{q} } \right) - { }} \right. \hfill \\ \left. {N_{q} \left( t \right)f_{{k^{\prime}}} \left( {1 - f_{k} } \right)\delta \left( {\varepsilon_{{k^{\prime}}} - \varepsilon_{k} - h\omega_{q} } \right)} \right\} \hfill \\ \end{gathered}$$

Here, the spin and the valley degeneracies are $g_{s} = 2$ and $g_{v} = 2$, respectively. $C_{q} = \sqrt {\left| \Lambda \right|^{2} q/2\rho  v_{s} }$, where $\Lambda$ is the deformation potential, ρ is the density of the graphene sheet, and $\tau$ is the relaxation constant. The factor $f_{k} \left( {1 - f_{k^{\prime}} } \right)$ is the probability that the initial state *k* is occupied and the final electron state *k*′ is empty. *f*_*k*_ is the unperturbed Fermi–Dirac distribution function. The factor $N_{q} f_{{k^{\prime}}} \left( {1 - f_{k} } \right)$ is that of the boson and fermion statistics, and $\varepsilon_{{k,k^{\prime}}}$ is the energy dispersion. With *A* being the area of the material, the summation in Eq. (1) spans over *k*, *k*′ and can be transformed into an integral as

2 $$\mathop \sum \limits_{{k,k^{\prime}}} \to \frac{{A^{2} }}{{\left( {2\pi } \right)^{4} }}\smallint d^{2} kd^{2} k^{\prime},$$

Considering $N_{q} \left( t \right) \gg 1$, yields $\frac{{\delta N_{q} }}{\delta t} = {\Gamma }_{q} N_{q}$, where, $\Gamma_{{\varvec{q}}}$ is the absorption coefficient, expressed as:

3 $$\Gamma_{q} = \frac{{A\left| {\Lambda } \right|^{2} q}}{{(2\pi)^{3} v_{f} \rho v_{s} }}\mathop \smallint \nolimits_{0}^{\infty } kdk\mathop \smallint \nolimits_{0}^{\infty } k^{\prime}dk^{\prime}\mathop \smallint \nolimits_{0}^{2\pi } d\varphi \mathop \smallint \nolimits_{0}^{2\pi } \left\{ {d\theta \left[ {f\left( k \right) - f\left( {k^{\prime}} \right)} \right]*} \right.\left. {\delta \left( {k - k^{\prime} - \frac{1}{{hv_{f} }}\left( {h \omega_{q} + v_{D} h q} \right)} \right)} \right\}$$

where $v_{f}$ is the Fermi velocity, $v_{s}$ is the velocity of sound, and $\hbar$ is Planck’s constant, *φ* is the angle between *k* and the z-direction, and *θ* is the angle between *k* and *k'*. The energy dispersion of *SLG* varies linearly with *k* and is given as $\varepsilon \left( k \right) = \pm \hbar v_{f} \left| k \right|$ ^53^. We first analyze the effect of temperature change on the mobility of electrons in the *SLG* by switching off the applied voltage. This enables the study of the effect of carrier concentration under various temperatures, a consequence of energy conservation in the electron–phonon scattering process, ${ }k^{\prime} = k - \frac{1}{{\hbar v_{f} }}\left( {\hbar \omega_{q} } \right)$. Considering the condition where $k_{B} T \ll 1,$ the Fermi–Dirac distribution becomes $f\left( k \right) = {\text{exp}}\left( { - \beta \left( {\varepsilon \left( k \right)} \right)} \right)$, where $\beta = 1/k_{B} T$ (*k*_*B*_ is the Boltzmann constant). The absorption coefficient relates to the *AE* current via the Weinrich^25^ relation, as follows:

4 $$J_{ac} = \frac{{2e \tau }}{{hv_{f} }}\Gamma_q$$

Thus, the acoustoelectric current ${J}_{ac}$ can be expressed as

5 $$J_{ac} = - \frac{{A|\Lambda |^{2} e\tau{q} }}{{ {2\pi } ^{3} (h v_{f})^{2} \rho v_{s} }}\int_{0}^{2\pi } {kdk(k - \frac{1}{{hv_{f} }}(h\omega_{q} ))} [exp( - \beta hv_{f} k) - exp( - \beta hv_{f} (k - + \frac{1}{{hv_{f} }}(h \omega_{q} )]$$

Integrating and simplifying Eq. (5) yields

6 $$J_{ac} = J_{0} \left\{ {2 - \beta \hbar \omega_{q} } \right\}\left[ {1 - {\text{exp}}( - \beta \hbar \omega_{q} )} \right]$$

where $$J_{0} = \frac{{2\tau A|\Lambda |^{2} kTq }}{{(2\pi )^{3} \beta^{3} h^{4} v_{f}^{4} \rho V_{s} }}$$.

**Figure 1:**
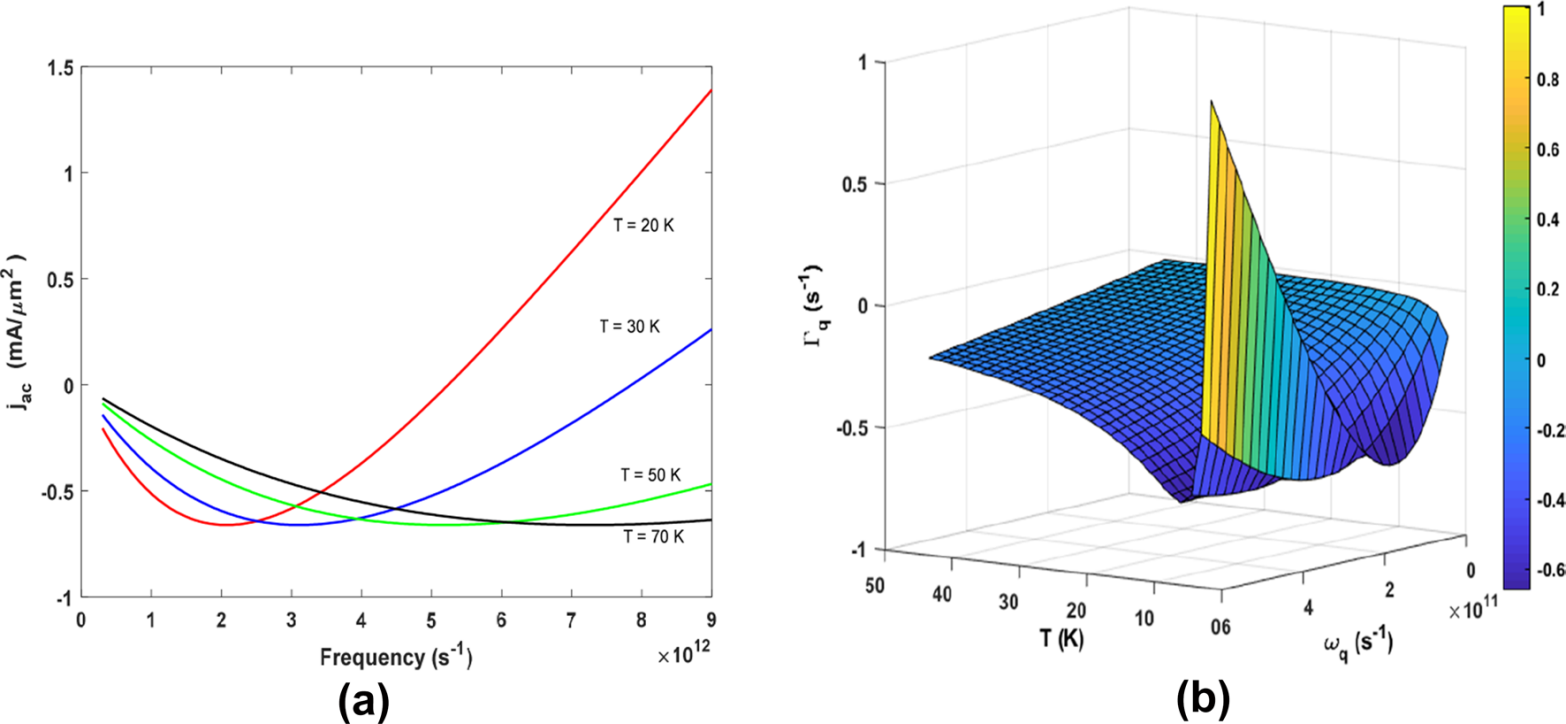
(**a**) Dependence of $J_{ac}$ on $\omega_{q}$ for various temperatures (*T*), and (**b**) a meshgrid 3D plot of ${\Gamma }_{q}$ versus $\omega_{q}$ and *T*.

From Eq. (6), $J_{ac}$ varies with temperature as $T^{4} ,$ which, according to Mariana and Von Oppen^58^ indicates the contribution of an in-plane acoustic phonon. In Fig. 1a, we show the dependence of $J_{ac}$ on frequency ($\omega_{q}$) at various temperatures (T = 20, 30, 50, and 70 K) using the following parameters, $v_{F} \approx 10^{8} {\text{ms}}^{ - 1}$, $\tau = 5*10^{ - 10} \;{\text{s}}$, $\Lambda = 9\;{\text{eV}}$, $V_{s} = 2.1 \times 10^{3} \;{\text{cm}}\;{\text{s}}^{ - 1}$ and q = 10^5^ cm^−1^. The plot shows a nonlinear *AE* current $J_{ac}$ which decreases with an increase in temperature. The *AE* current does not exhibit a simple linear dependence on $\omega_{q} ,$ and temperature. At T = 20 K, the current initially decreased to a minimum at 2 *THz*, and then increased at higher frequencies. A similar trend was observed when the temperature was increased to 30K. However, at 50K and 70K, the increase in $J_{ac}$ is gradual, with turning points at 5 THz and 8 THz, respectively. Thus, in general, increasing the temperature decreases the current. This indicates the transport of holes in the material, and as the temperature increases, the lattice vibration also increases, limiting the flow of the acoustoelectric current. From the relation $I = \hbar \omega_{q}$, the intensity of the acoustic phonons is directly proportional to the frequency $(\omega_{q} ).$ Thus, Fig. 1a is qualitatively in agreement with the experimental work of Bandhu and Nash (see Fig. 4a^34^), where they measured the acoustoelectric current for several temperatures at various frequencies in the MHz region. However, in this study, the frequencies are in the THz region.

**Figure 2:**
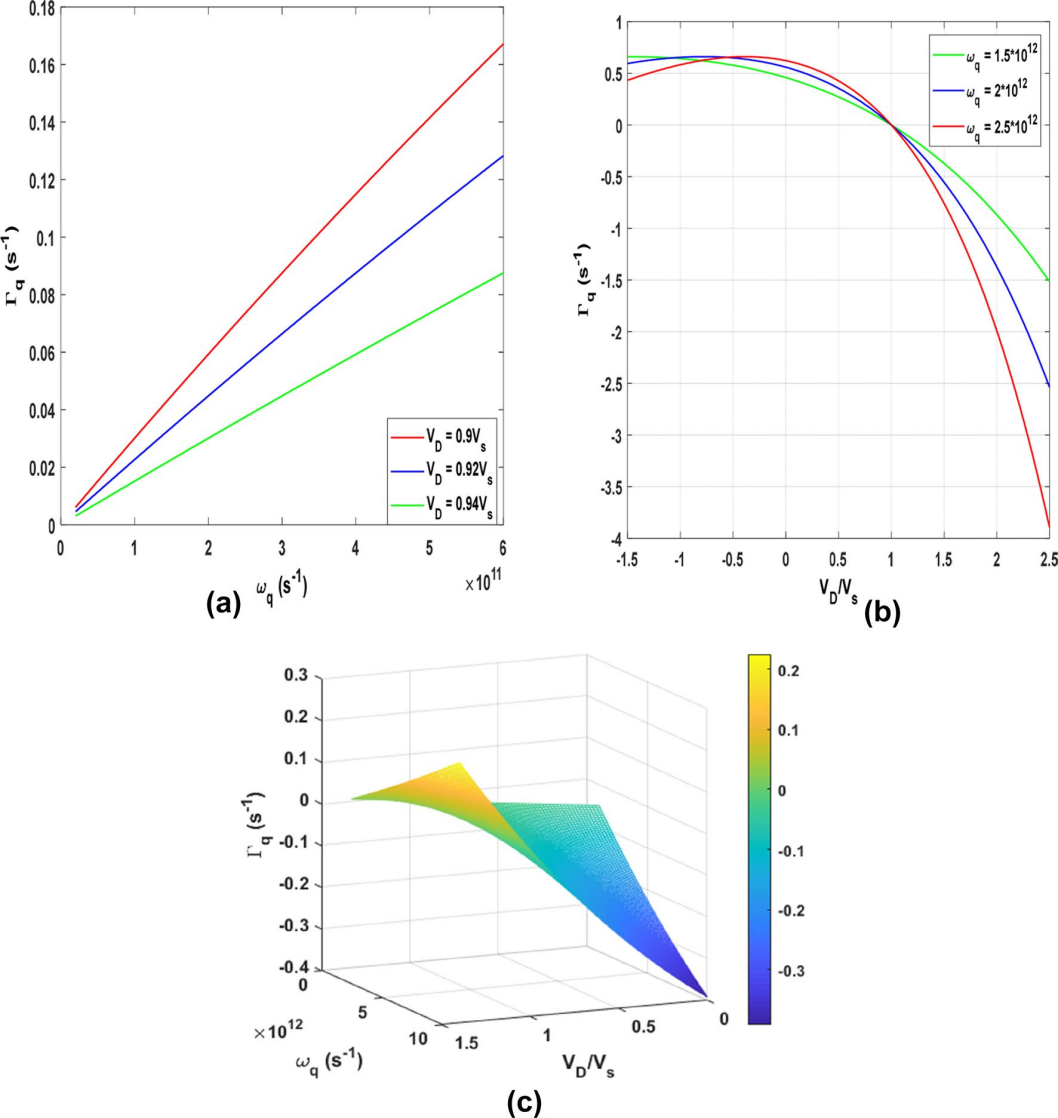
(**a**) Dependence of $\Gamma_{q}$ on $\omega_{q}$ for *v*_D_ = 0.9*v*_*s*_, 0.92*v*_*s*_, and 0.94*v*_*s*_, and (**b**) the dependence of ${\Gamma }_{q}$ on $v_{D} /v_{s}$ at varying $\omega_{q}$. = 1.2, 2, 2.2 THz. (**c**) A 3D plot of the dependence of ${\Gamma }_{q}$ on $v_{D} /v_{s}$ and $\omega_{q}$.

To further illustrate this, the simultaneous dependence of the ${\Gamma }_{q}$ on frequency ($\omega_{q}$) and temperature (*T*) is shown as a 3D plot in Fig. 1b. For the dependence of ${\Gamma }_{q}$ on *T*, the graph decreased to a minimum and then increased to a point and remained constant at higher temperatures, while the dependence of ${\Gamma }_{q}$ on $\omega_{q}$ conformed to that of Fig. 1a. By switching on the drift field $k^{\prime} = k - \frac{1}{{\hbar v_{f} }}\left( {\hbar \omega_{q} + v_{D} .\hbar q} \right)$, Eq. (3) becomes

7 $${\Gamma }_{{\varvec{q}}} = {\Gamma }_{0} \left\{ {2 - \beta \hbar \omega_{q} \left( {1 - \frac{{v_{D} }}{{v_{s} }}} \right)} \right\}\left[ {1 - {\text{exp}}\left( { - \beta \hbar \omega_{q} \left( {1 - \frac{{v_{D} }}{{v_{s} }}} \right)} \right)} \right]$$

where $\Gamma_{0} = J_{0}$. Then, Eq. (7) can be numerically analysed for a normalized ${\Gamma }_{q}$ dependence on $v_{D} /v_{s}$ and $\omega_{q}$. Shown in Fig. 2a is the dependence of ${\Gamma }_{q}$ on $\omega_{q}$ for v_D_ = 0.9*v*_*s*_, 0.92*v*_*s*_ and 0.94*v*_s_, which depicts a linear relationship. However, Γ_*q*_ decreases when *v*_D_ increases.

**Figure 3:**
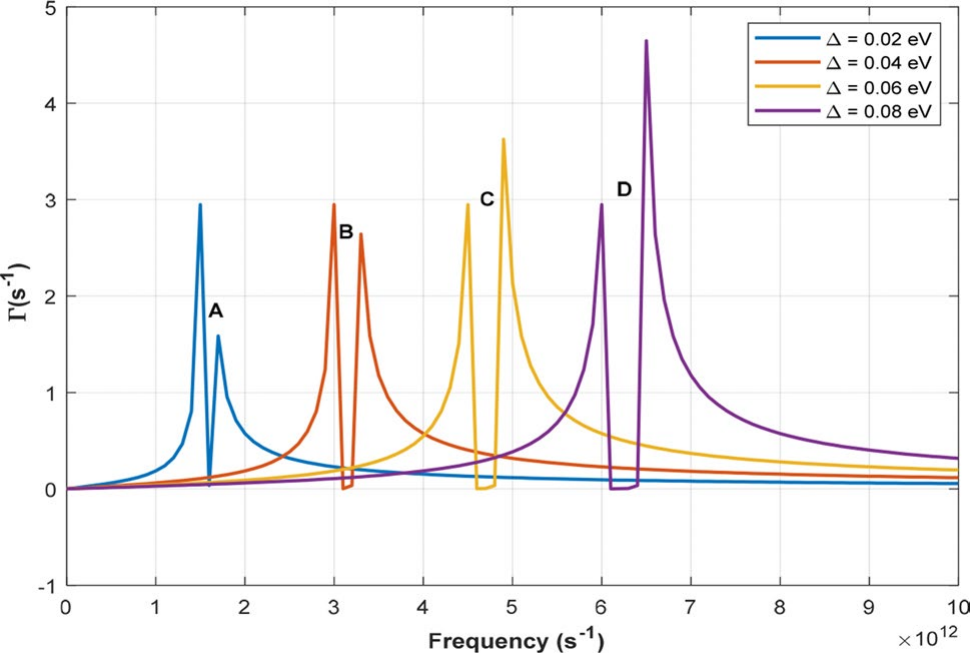
Absorption spectra showing the dependence of ${\Gamma }_{q}$ on the $\omega_{q}$. The gap broadens as the energy gap ($\Delta$) increases.

The Weinreich relation $J_{ac} = - \frac{2e\tau }{{\hbar v_{f} }}{\Gamma }_{{\varvec{q}}}$ relates the absorption to *AE* current. Thus, Fig. 2a is qualitatively in agreement with a previous experimental report (see Fig. 3^57^), where the *AE* current varied linearly with the frequency. Figure 2b shows the dependence of ${\Gamma }_{q}$ on $v_{D} /v_{s}$ for various values of $\omega_{q}$ when a non-quantizing electric field is applied along the axis of the $SLG$. Absorption and amplification occur when $v_{D} /v_{s} < 1$ and $v_{D} /v_{s} > 1$, respectively, which is consistent with the work of Nunes and Fonseca^29^. In Fig. 2c, we show a 3D graph of the dependence of ${\Gamma }_{q}$ on $v_{D} /v_{s}$ and $\omega_{q}$. Setting $v_{D} = 1.1v_{s}$, the maximum amplification is obtained at ${\Gamma }_{q}$ =  − 0.16 for $\omega_{q}$ = 2 THz. It is interesting to note that, our results are in good agreement with the work of Bandhu et al.^57^, where acoustic-phonon frequencies above 10 THz were attained. The field *E* in the *SLG* can be calculated using $E_{D} = v_{D} /\mu$, where $\mu = 2.0 \times 10^{4} \;{\text{cm}}^{2} /v_{s}$ is the electron mobility in graphene. Using $v_{s} = 2.1 \, \times 10^{5} \;{\text{cm/s}}$ gives $E_{D} = 11.5\;{\text{V/cm}}$. For the source-to-drain voltage, $V_{sd} = v_{D} L/\mu$, (*L* is the length from the source to the drain electrode in the graphene), the in-plane current $I = env_{D} L$ (*n* is the electron density) can be calculated.

Patterning *SLG* into $GNR$ opens a band gap $\left( \Delta \right)$^54^ with the energy dispersion given by^53^

8 $$\varepsilon (k) = \frac{\Delta }{2}\sqrt {1 + \frac{{\hbar^{2} k^{2} }}{{\left( {\hbar B} \right)^{2} }}}$$

where Δ is the energy gap and *B* is the quantized wave vector. By considering that the acoustic phonon and the electric field are directed along the *GNR* axis, $k^{^{\prime}} = (k + \hbar q)\cos \theta$, where $\theta$ is the scattering angle. When a field is applied to the *GNR*, the energy level degenerates. At low temperatures, when $\varepsilon (p) \gg \hbar \omega_{q}$, Eq. (1) becomes (see Supplementary information)

9 $$\Gamma_{q} = - \frac{{\pi C_{q}^{2} \Delta \hbar q}}{{4\hbar^{2} B^{2} }}\left\{ {I_{1}^{{ - \frac{1}{2}}} *\alpha - I_{2}^{{ - \frac{1}{2}}} *\beta } \right\}*\left( {\sqrt {4\left( {2\left( {\hbar B} \right)^{2} - \frac{{2\hbar^{4} \omega_{q}^{2} B^{2} }}{{\Delta^{2} }}\left( {1 - \frac{{v_{D} }}{{v_{s} }}} \right)^{2} } \right)} } \right)^{ - 1}$$

where

$$I_{1} = \left[ {1 + \frac{1}{{4\left( {\hbar B} \right)^{2} }}\left( { - \hbar q\cos \left( \theta \right) + \sqrt {4\left( {2\left( {\hbar B} \right)^{2} - \frac{{2\hbar ^{4} \omega _{q}^{2} B^{2} }}{{\Delta ^{2} }}\left( {1 - \frac{{v_{D} }}{{v_{s} }}} \right)^{2} } \right)} } \right)^{2} } \right]$$

$$\alpha = - \hbar qcos\left( \theta \right) + \sqrt {4\left( {2\left( {\hbar B} \right)^{2} - \frac{{2\hbar^{4} \omega_{q}^{2} B^{2} }}{{\Delta^{2} }}\left( {1 - \frac{{v_{D} }}{{v_{s} }}} \right)^{2} } \right)}$$

$$I_{2} = \left[ {1 + \frac{1}{{4\left( {\hbar B} \right)^{2} }}\left( { - \hbar q\cos \left( \theta \right) - \sqrt {4\left( {2\left( {\hbar B} \right)^{2} - \frac{{2\hbar ^{4} \omega _{q}^{2} B^{2} }}{{\Delta ^{2} }}\left( {1 - \frac{{v_{D} }}{{v_{s} }}} \right)^{2} } \right)} } \right)^{2} } \right]$$

$$\beta = \left( { - \hbar qcos\left( \theta \right) - \sqrt {4\left( {2\left( {\hbar B} \right)^{2} - \frac{{2\hbar^{4} \omega_{q}^{2} B^{2} }}{{\Delta^{2} }}\left( {1 - \frac{{v_{D} }}{{v_{s} }}} \right)^{2} } \right)} } \right)$$

In Eq. (9), $\left( {\hbar B} \right)^{2}$ is the quantized acoustic phonon energy, where $B = \frac{2\pi }{{a_{c - c} \sqrt 3 }}\left( {\frac{Pi}{{N + 1}} - \frac{2}{3}} \right)$, *N* is the width of the graphene, $a_{c - c}$ is the lattice constant, and *Pi* is the sub-band index. The absorption reveals the characteristic feature of the acoustic phonon spectrum in the materials that occurs in the Terahertz frequency range. In addition to the parameters used in Fig. 1, the following are used: $N \approx 500\;{\text{nm}}$, $\Delta = 0.02,0.04,0.06,0.08\;{\text{eV}}$, and $v_{D} < v_{s}$. The plot of ${\Gamma }_{q}$ versus $\omega_{q}$ in Eq. (9) is shown in Fig. 3, which depicts a twin peak with a varying peak heights. The gap between them shifts to the right as the frequency increases. This is similar to the experimental report by Wu (see Fig. 5a^54^). The twin peaks occur as a result of electron transport in the dual-band formed in the *GNR.* In the first band, the electrons are initially absorbed until they encounter a gap, where they lose their energy. They then gains energy in the second band by absorbing the energy of the confined phonons. This occurs at low drift velocities of $v_{D} = 0.1v_{s}$ where the electron energy is comparable to the band gap energy. The peak difference is due to a change in the Fermi energy.

The first gap occurs at $\Delta = 0.02$ eV while the second, the third and the fourth occur at 0.04, 0.06, 0.09 eV, respectively. From the plot, gaps occur at points where the ${\Gamma }_{q} = 0$. At A, we obtained a partial gap, but B, C, and D showed a complete gap. When, ${\Gamma }_{q} = 0$, from Eq. (9), we obtain

10 $$\Delta = \hbar \omega_{q} \left[ {1 - \frac{{v_{D} }}{{v_{s} }}} \right]$$

**Figure 4:**
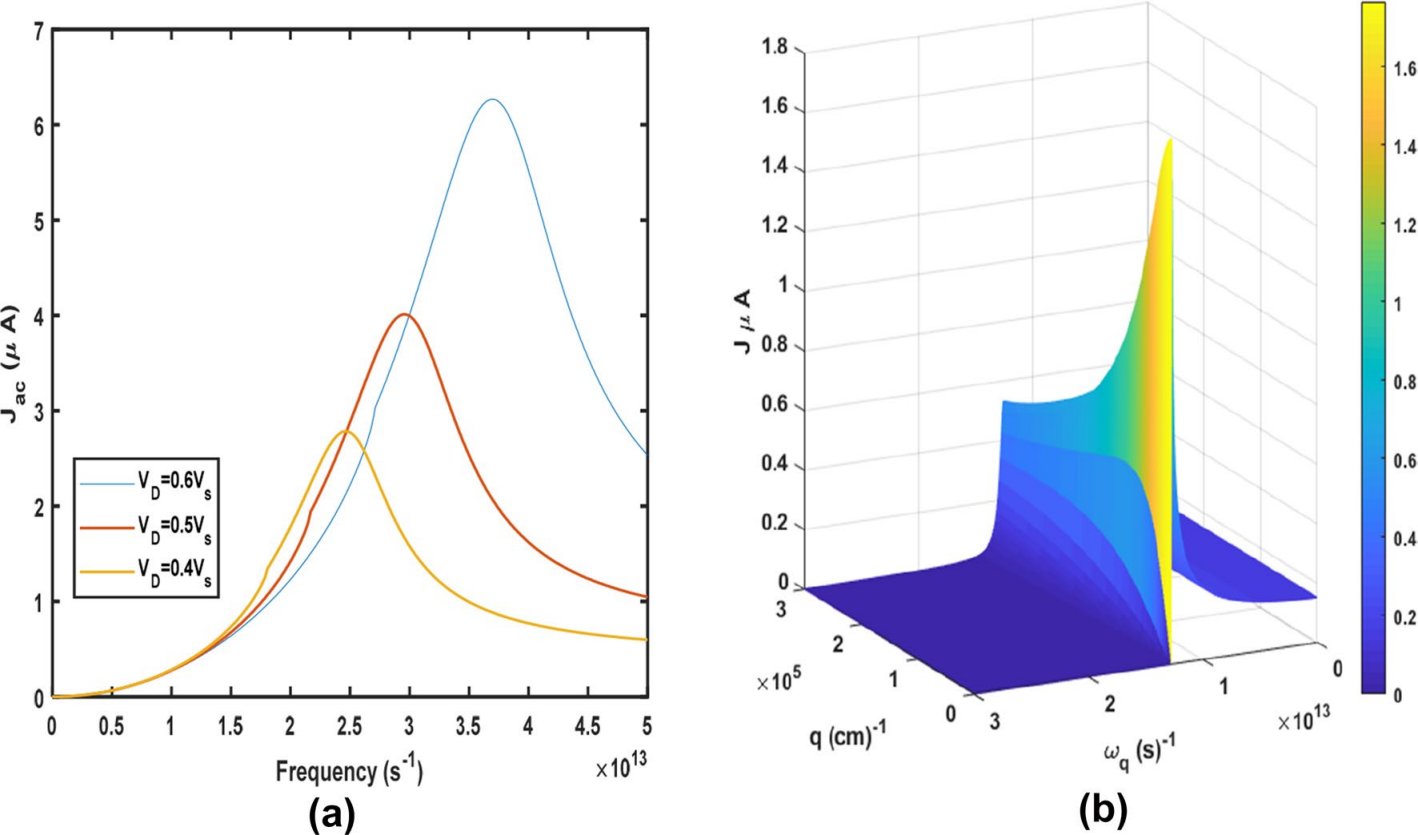
(**a**) Dependence of $J_{ac}$ on $\omega_{q}$ at various drift velocities $v_{D}$, and (**b**) a 3D plot of the dependence of $J_{ac}$ on $\omega_{q}$ and *q*.

Therefore, knowing, $v_{D}$, $v_{s}$ and $\omega_{q}$, the energy gap ($\Delta$) of the material can be determined as in Eq. (10). Using the Weinreich relation and Eq. (9) Fig. 4a shows the acoustoelectric versus ${\omega }_{q}$ at drift velocities of ${v}_{D}=0.4{v}_{s}, 0.5{v}_{s}, 0.6{v}_{s}$. The current increases to a maximum point (resonant) and then decreases. At these drift velocities, the energy of the electron is able to overcome the bandgap energy. The resonant point is referred to as the threshold frequency ${\omega }_{q}^{TH}$, beyond which the current decreases. The resonance peak is dependent on $v_{D}$. The plot shifts to the right when $v_{D}$ is increased, making $AE$ in graphene tunable. Figure 4a is qualitatively consistent with the experimental report by Poole et al. (see Fig. 3^35^). In Figs. 3 and 4, the conduction mechanism is via intraband transitions. Unlike Fig. 3, for a certain quantized phonon energy, the absorption ${\Gamma }_{q} = 0$ spectrum in Fig. 4a is due to conduction electrons crossing the energy gap at higher drift velocities and subsequently absorbing the energy of the confined phonons. For further elucidation, a 3D plot of $J_{ac}$ versus $\omega_{q}$ and *q* is shown in Fig. 4b. Similar results were obtained experimentally in the Megahertz (MHz) range by Liag et al.^48^, Okuda et al.^50^, and Morgado et al.^51^. In the Terahertz range, the simulated results of absorption in graphene obtained by Ullah et al.^55^ are in qualitatively agreement with Fig. 4.

The $LDOAP$ could be achieved by stimulating the GNR with THz radiation under a gated voltage to modulate the carrier concentration in the GNR. The unique band structure permits absorption via intraband electronic transitions, which can be used to adjust the electron density in the material. The carrier density can be controlled easily and efficiently by varying the gate voltage. The field $E$ in the *SLG* can be calculated by using $E = {v}_{D}/\mu$, where $\mu = 2.0 \times 10^{4} \;{\text{cm}}^{2} /v_{s}$ is the electron mobility in graphene. Using $v_{s} = 2.1 \, \times 10^{5} \;{\text{cm/s}}$ gives $E = 11.5 \;{\text{V/cm}}$. For the source-to-drain voltage, $V_{sd} = { }v_{D} {\text{L}}/{\upmu }$, (*L* is the length from the source to the drain electrode in the graphene), the in-plane current $I = env_{D} L$ (*n* is the electron density) can be calculated.

## Conclusion

We have theoretically demonstrated the acoustoelectric current generation in graphene nanoribbon resulting from Landau damping of acoustic phonons. The AE current in a single layer of graphene was calculated when the temperature and the electric fields were applied. Graphene nanoribbon exhibited a larger acoustoelectric current than a single layer of graphene when a non-quantizing field was applied. The acoustoelectric current shifts as the drift velocity is varied. This makes acoustoelectric current in graphene nanoribbon tunable and a good acoustic wave filter for phonon spectroscopy.

## Supplementary Information

Supplementary Information.
