# Supplementary material for: Acoustoelectric current in graphene nanoribbon due to Landau damping
Source: Sci Rep. 2021 Sep 9;11:17913. doi: 10.1038/s41598-021-95896-6 (PMC8429432; doi:10.1038/s41598-021-95896-6)
Supplement: Supplementary file 1 — Supplementary Information. [file 41598_2021_95896_MOESM1_ESM.docx]

**Supplementary Information**

The derivative of the Fermi-Dirac distribution is a $\delta-$function at $E_{F}=\vartheta$ (chemical potential), given as

$f(\varepsilon(k+q))-f(\varepsilon(k))=\frac{\partial f(\varepsilon_{k})}{\partial k}$ (SI-1)

The , where $\frac{\partial f(\varepsilon_{k})}{\partial\varepsilon_{k}}=-\Theta\left(\varepsilon_{F}-\varepsilon_{k}\right)$and . Eqn. (SI-1) can further be expressed as:

(SI-2)

Considering $\sum_{k} \to\frac{1}{\left(2\pi\right)^{2}}\int dk$, and substituting Eqn. (SI-2) into Eqn. (1) yields:

(SI-3)

Where $\Theta(E_{F}-\varepsilon(k)):=\left\{_{0,\text{ E}_{F}<\varepsilon(k)}^{1,\text{ E}_{F}>\varepsilon(k)}\right.$. Via the energy conservation principle, yields

Taking the root of the quadratic equation gives

(SI-4)

substituting Eqn. (SI-4) into Eqn.(SI-3) and using the standard identity $\int_{-\infty}^{\infty} f\left(x\right)\delta\left(g\left(x\right)\right)dx=\sum_{i} \frac{f\left(x_{i}\right)}{g'\left(x_{i}\right)}$, and considering

Eqn. (SI-3) can be solved after cumbersome calculations to yield

*

(SI-5)
